# Evaluation of mandibular bone abnormalities in patients with chronic kidney disease using cone beam computed tomography: A retrospective study

**DOI:** 10.2340/aos.v84.44619

**Published:** 2025-09-03

**Authors:** Heelim Lee, Jae Suk Jung, Jiwoo Lee, Seung Il Song, Jeong Keun Lee, Ji Min Kim, Bumhee Park, Inwhee Park, Suk Ji

**Affiliations:** a Department of Periodontology, Institute of Oral Health Science, Ajou University School of Medicine, Suwon, Korea; b Department of Oral and Maxillofacial Surgery, Institute of Oral Health Science, Ajou University School of Medicine, Suwon, Korea; c Office of Biostatistics, Medical Research Collaborating Center, Ajou Research Institute for Innovative Medicine, Ajou University Medical Center, Suwon, Republic of Korea; d Department of Nephrology, Ajou University School of Medicine, Suwon, Korea

**Keywords:** Renal insufficiency, chronic, bone diseases, metabolic, cone-beam computed tomography, dental implantation

## Abstract

**Objective:**

This study evaluated mandibular cortical thickness and morphological changes in chronic kidney disease (CKD) patients using cone beam computed tomography (CBCT) and their correlation with bone metabolism markers.

**Methods:**

CKD patients were divided into CKD-I (eGFR (estimated glomerular filtration rate) < 30) and CKD-II (eGFR ≥ 30) groups, with healthy controls for comparison. Mental index (MI), antegonial index (AI), and panoramic mandibular index (PMI) were compared using Kruskal-Wallis test. Mandibular cortical index (MCI) classifications, lamina dura loss, and soft tissue calcifications were assessed using Fisher’s test. Relationships between serum bone metabolism markers and radiomorphometric indices were analyzed by linear regression.

**Results:**

The study included 94 CKD patients (56 CKD-I, 38 CKD-II) and 88 controls. MI and AI were significantly lower in CKD-I versus controls (*p* < 0.05). MCI class I was less prevalent in CKD-I (5.4%) than controls (35.2%) (*p* < 0.001). Lamina dura loss (*p* = 0.006) and soft tissue calcifications (*p* = 0.009) occurred more frequently in CKD groups. Elevated alkaline phosphatase was associated with reduced cortical thickness (*p* < 0.01).

**Conclusions:**

Although findings should be interpreted considering the retrospective design’s limitations, CBCT revealed significant bone abnormalities in CKD patients, with compromised bone quality and reduced mandibular cortical thickness, especially in advanced renal impairment, suggesting its value for pre-implant bone quality assessment in CKD patients.

## Introduction

Chronic kidney disease (CKD) is a global health concern with rising prevalence affecting an estimated 800 million patients worldwide in 2017 [1]. As the incidence of CKD increases, dentists are increasingly likely to encounter patients with this disease when performing bone surgery, including implant surgery.

When kidney function deteriorates due to CKD, bone mineral metabolism is disrupted, and the homeostasis of calcium (Ca) and phosphate (P) is altered. As the disease progresses, these effects are exacerbated, potentially leading to renal osteodystrophy, which further affects bone metabolism as a result of CKD and secondary hyperparathyroidism [2]. The collective term CKD-mineral and bone disorder (CKD-MBD) encompasses various changes such as renal osteodystrophy, soft tissue calcification, and abnormalities in bone mineral metabolism [3]. Subsequently, the jaw bones are affected by CKD-MBD. This can have major clinical complications for osseointegration and survival of dental implants and the success of regeneration therapy [4]. A recent study showed that residual alveolar bone volume in dialysis patients was often sufficient for implant placement [5], and a recent systematic review showed that early implant placement in patients with CKD is clinically effective with implant outcomes similar to immediate and delayed placement protocols [6]. Nonetheless, concerns persist regarding the success of dental implants in individuals with CKD-MBD because optimal bone metabolism is critical for the success and long-term maintenance of dental implant procedures [5, 7, 8].

In the preparation for implant surgery, assessing bone stability and evaluating treatment outcome risks are critical. Dental cone beam computed tomography (CBCT) offers greater reliability and accuracy than panoramic radiography for detecting intra-bone lesions [9] and near-edge lesions [10], making it an essential tool for preoperative diagnosis and treatment planning [11]. However, a significant literature gap exists regarding quantitative assessment of mandibular bone changes in CKD patients using such advanced imaging techniques, with limited CBCT-based morphometric data available for evaluating bone quality in this population.

This study addresses this research gap through a retrospective observational investigation utilizing CBCT to evaluate mandibular bone abnormalities in CKD patients. We hypothesize that advanced CKD is associated with detectable mandibular bone abnormalities that correlate with biochemical bone metabolism markers. Our objectives were to assess bone abnormalities using specific CBCT-derived radiomorphometric indices, characterize these abnormalities based on disease severity, and determine correlations between radiographic findings and systemic factors, including bone markers. These findings will provide clinicians with valuable preoperative assessment tools for implant treatment planning in CKD patients, potentially improving surgical outcomes and patient care.

## Materials and methods

### Subjects

**Figure 1:**
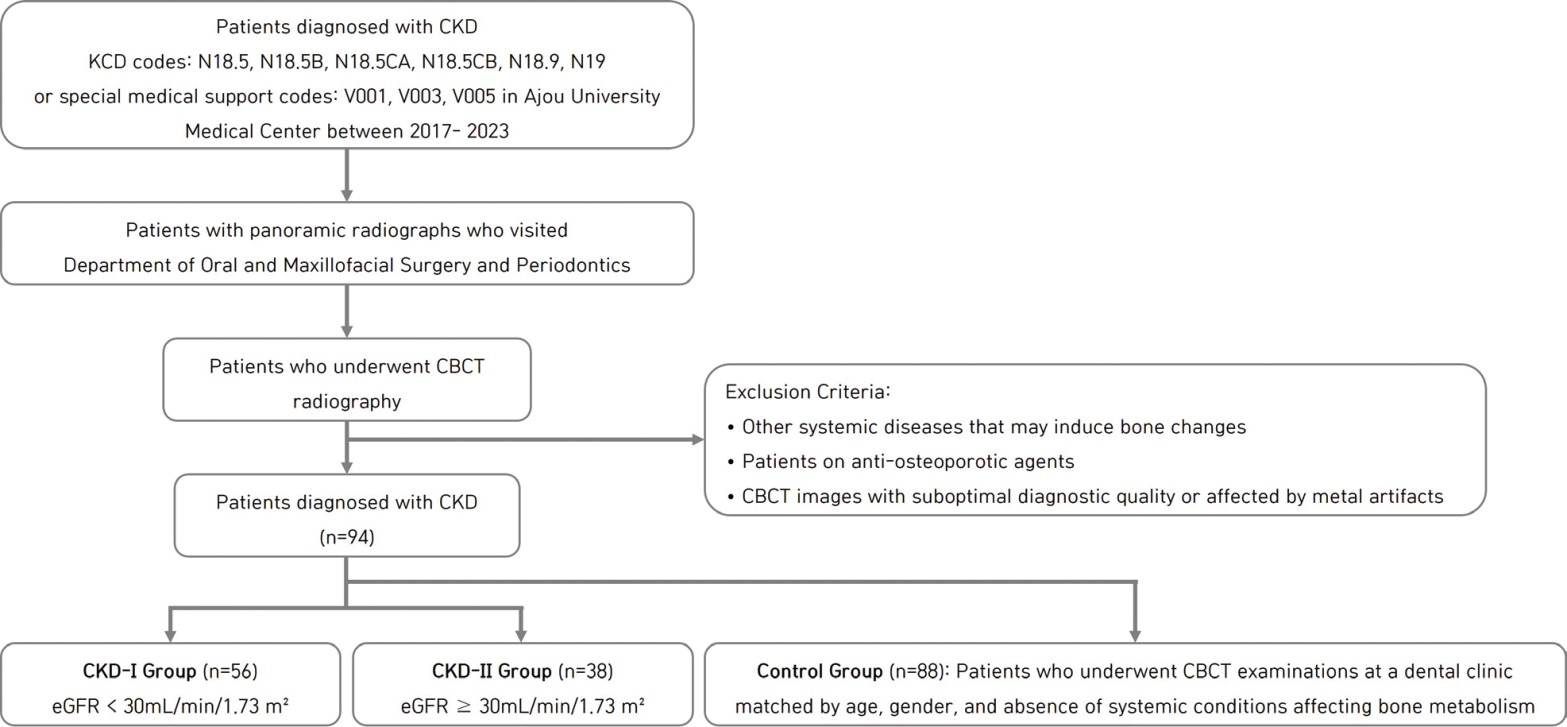
Flow chart outline of the patient selection and exclusion process.

This retrospective observational case-control study was approved by the Institutional Review Board (IRB) of Ajou University Medical Center (AUMC), under IRB File No. AJOUIRB-DB-2023-176. Patients who visited the medical center in Suwon, South Korea from January 1, 2017 to March 1, 2023 were screened for eligibility. We included those diagnosed with CKD based on the Korean Standard Classification of Diseases (KCD) codes N18.5, N18.5B, N18.5CA, N18.5CB, N18.9, and N19. Additionally, some of these patients were identified using special medical support codes for kidney replacement therapy (KRT): V001 for hemodialysis, V003 for peritoneal dialysis, and V005 for kidney transplantation. Among CKD patients, those with panoramic radiographs who visited AUMC Dental Hospital’s Department of Oral and Maxillofacial Surgery and Periodontics were first sorted. Among these patients, those who underwent CBCT radiography were selected for the test groups. Those with other systemic diseases, procedures, and medications that may induce bone changes (osteoporosis, thalassemia, autoimmune rheumatic diseases, radiotherapy, Paget’s disease, bisphosphonate medication, hyperthyroidism, and multiple myeloma) and those who were on anti-osteoporotic agents were excluded. The patient groups were stratified based on eGFR calculated using the Modification of Diet in Renal Disease formula. The eGFR threshold of 30 mL/min/1.73 m² was selected based on established KDIGO guidelines for CKD classification [3], which distinguishes between moderate-to-severe (eGFR < 30) and mild-to-moderate (eGFR ≥ 30) kidney dysfunction. This cutoff is clinically relevant as patients with eGFR <30 typically exhibit more severe MBDs [3, 12]. One subgroup had eGFRs < 30 mL/min/1.73 m² (CKD-I), and the other had eGFRs ≥ 30 mL/min/1.73 m² (CKD-II). The control group was selected from patient records of individuals who underwent CBCT examinations at a dental clinic for the purposes of third molar extraction or dental implant surgery. Controls were matched to the experimental group based on age, gender, and absence of systemic conditions affecting bone metabolism. Specific exclusion criteria for controls included: any history of kidney disease, diabetes mellitus, hyperthyroidism, osteoporosis, thalassemia, autoimmune rheumatic disease, cancer, smoking history, or use of medications affecting bone metabolism (Figure 1).

### CBCT image acquisition and radiographic measurements

**Figure 2:**
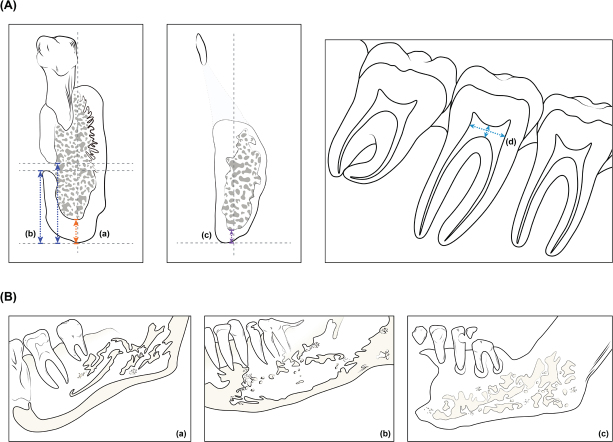
The measurements on CBCT: (a) The mental index (MI, orange double-ended arrow) was assessed at the middle of the mental foramen from a coronal view, and the cortical thickness of the mandible was measured along a line perpendicular to the mandible margin. (b) Panoramic mandibular index (PMI, blue double-ended arrow) was measured at the middle of the mental foramen from a coronal view, and the distance between the superior (PMI superior) and inferior (PMI inferior) margin of mental foramen and the inferior margin of mandible were measured and were divided by the thickness of the cortical part of mandible. (c) Antegonial index (AI, purple double-ended arrow) was measured in CBCT coronal view slices, and cortical thickness was measured from the antegonial notch area. (d) Pulp chamber size was measured on the CBCT panoramic view slices. Vertical and horizontal size measurements of pulp chambers of mandibular first molars (sky blue double-ended arrow) were performed on the narrowest part. Mandibular cortical index (MCI) classification: (a) MCI class I if both sides of mandible showed clear and sharp endosteal margin, (b) Class II if one or both sides of mandibular margin showed cortical residues and/or showed semilunar defects, and (c) Class III if the endosteal margin showed clearly porous margin and heavy residues.

Panoramic radiographs were obtained using CS 8100 (Carestream Dental, Atlanta, GA), and CBCT images were obtained using Dinnova3 (HDX, Seoul, Korea). All CBCT images were acquired with standardized parameters: voltage 110 kV, current 3–5 mA, voxel size 0.16 mm, exposure time 5.4 s, and slice thickness 0.25 mm. A metal artifact reduction (MAR) algorithm was applied to all scans to minimize interference from dental restorations. The device was calibrated weekly according to manufacturer specifications, and quality assurance tests were performed monthly. Images were reconstructed using OnDemand software (Cybermed, Seoul, Korea). A single calibrated examiner, blinded to participant group assignment and clinical data, performed all measurements. Measurements from Ledgerton’s classification [13] were obtained from the CBCT radiography. The mental index (MI) was assessed at the middle of the mental foramen from a coronal view, and the cortical thickness of the mandible was measured along a line perpendicular to the mandible margin (Figure 2Aa). The panoramic mandibular index (PMI) was measured from the same cut, and the distances between the superior and inferior margin of the mental foramen and the inferior margin of the mandible were measured. These were divided by the thickness of the cortical part of the mandible (Figure 2Ab). The antegonial index (AI) was measured in CBCT coronal view slices, and cortical thickness was measured from the antegonial notch area (Figure 2Ac). Mandibular cortical index (MCI) classifications were identified on panoramic view slices and sagittal view slices on which endosteal margins of mandibular cortex were examined. Each class was evaluated: (1) MCI class I if both sides of the mandible showed clear and sharp endosteal margins, (2) Class II if one or both sides of the mandibular margin showed cortical residues and/or semilunar defects, and (3) Class III if the endosteal margin showed a clearly porous margin and heavy residues (Figures 2Ba–c). Pulp chamber size measurements were included based on previous research demonstrating that CKD-related MBDs can affect dental hard tissues beyond alveolar bone [14]. Vertical and horizontal dimensions of mandibular first molar pulp chambers (#36 or #46) were measured at the narrowest part on CBCT panoramic view sections (Figure 2Ad). Soft tissue calcifications were reviewed from CBCT axial view slices, and cases such as carotid artery calcifications (CAC), osteoma cutis (OC), sialolith (SL), tonsillolith (TL), and pulpal stones in pulp chambers were detected. These cases were reviewed in different CBCT cuts. Lamina dura loss was identified on CBCT panoramic view slices and panoramic radiographs.

### Collection of markers related to bone metabolism in serum

Data from serum marker levels related to bone metabolism were obtained from the medical records of test and control group subjects. These data included ALP (Alkaline phosphatase) level (IU/L); Ca level (mg/dL); P level (mg/dL); PTH (Parathyroid hormone) intact electrochemiluminescence immunoassay (ECLIA) results (pg/mL); thyroid-stimulating hormone (TSH) level (µlU/mL); and serum vitamin D,25(OH)D and 1,25(OH)_2_D, levels (ng/mL). Test values obtained on the date closest to that on which CBCT images were taken were used for analysis. These values were required to have been obtained within a 6-month period.

### Statistical analysis

Statistical analysis was performed using R software version 4.1.2. For continuous variables (age, MI, PMI, AI, pulp chamber size), the Kruskal-Wallis test was used for overall group comparisons, followed by the Mann-Whitney U test with Bonferroni correction for pairwise comparisons. Categorical variables (sex, KRT types, medications, systemic diseases, MCI, lamina dura loss, soft tissue calcifications) were analyzed using Fisher’s exact test with Bonferroni correction for multiple comparisons. Linear regression analysis was performed to assess associations between bone abnormalities and influencing factors (biochemical markers, CKD duration, KRT exposure time, sex, and age). Variables with *p* < 0.10 in univariate analysis were included in multivariate models. Multicollinearity was assessed using variance inflation factors, with all VIF values <2.5 confirming no significant multicollinearity among predictor variables. Statistical significance was set at *p* < 0.05.

## Results

### Demographic and clinical characteristics of study participants

Test and control groups consisted of 94 patients and 88 subjects, respectively. The CKD-I subgroup with eGFR < 30 mL/min/1.73 m² and the CKD-II subgroup with eGFR ≥ 30 mL/min/1.73 m² were composed of 56 and 38 patients, respectively. Of the 94 patients with CKD, 68 were receiving KRT, with hemodialysis accounting for the largest proportion at 44 patients. 93.2% of patients receiving hemodialysis (code V0001) were included in CKD-I, whereas 80.8% of patients not receiving KRT were included in the CKD-II group. There were 48 patients diagnosed with diabetes mellitus in the CKD group (Table 1).

**Table 1:** Demographic and clinical characteristics of study participants.

Parameter	Patients with CKD (*n* = 94)		Control (*n* = 88)	*P*
	CKD-I (eGFR < 30) (*n* = 56)	CKD-II (eGFR ≥ 30) (*n* = 38)		
Age at index date (*M* ± SD)	58.4 ± 16.4	58.9 ± 14.3	57.9 ± 15.9	0.964
18–29, *n* (%)	4 (7.1)	2 (5.3)	7 (8.0)	
30–39, *n* (%)	3 (5.4)	1 (2.6)	4 (4.6)	
40–49, *n* (%)	9 (16.1)	7 (18.4)	15 (17.1)	
50–59, *n* (%)	14 (25.0)	7 (18.4)	20 (22.7)	
60–69, *n* (%)	11 (19.6)	12 (31.9)	21 (23.9)	
≥ 70, *n* (%)	15 (26.8)	9 (23.7)	21 (23.9)	
Sex (male), *n* (%)	30 (53.6)	19 (50.0)	46 (52.3)	0.962
Kidney replacement therapy				< 0.001^a^
Code V001 (hemodialysis), *n* (%)	41 (93.2)	3 (6.8)		
Code V003 (peritoneal dialysis), *n* (%)	4 (57.1)	3 (42.9)		
Code V005 (kidney transplant), *n* (%)	6 (35.3)	11 (64.7)		
Non-kidney replacement therapy, *n* (%)	5 (19.2)	21 (80.8)		
Medication				< 0.001^a^
Steroid, *n* (%)	9 (16.1)	11 (28.9)	2 (2.3)	0.017^a^
Vitamin D and its analog, *n* (%)	25 (44.6)	7 (18.4)	2 (2.3)	0.003^a^
Phosphate binder, *n* (%)	16 (28.6)	0	0	<0.001^a^
Anti-depressant, *n* (%)	2 (3.6)	1 (2.6)	2 (2.3)	0.002^a^
Opioids, *n* (%)	1 (1.8)	0	0	1.000
Gabapentinoids, *n* (%)	4 (7.1)	3 (7.9)	2 (2.3)	0.013^a^
Systemic disease other than CKD				
DM, *n* (%)	27 (48.2)	21 (55.3)	0	< 0.001^a^
Hyperlipidemia, *n* (%)	2 (3.6)	3 (7.9)	5 (5.7)	-
Cardiovascular disease, *n* (%)	1 (1.8)	0	6 (6.8)	-
Cerebrovascular disease, *n* (%)	1 (1.8)	1 (2.6)	5 (5.7)	-
Other (psychological, allergy), *n* (%)	2 (3.6)	0	5 (5.7)	-

*M* ± SD: mean ± standard deviation; *n* (%): number (%); CKD: chronic kidney disease; eGFR: glomerular filtration rate; DM: diabetes mellitus.

a Statistically significant difference using Fisher’s exact test (*p* < 0.05).

### CBCT image measurements: A comparative analysis

MI measurements revealed significant cortical thinning in CKD-I patients bilaterally (Right: 2.9 ± 0.7 mm, Left: 2.9 ± 0.7 mm) compared to controls (Right: 3.2 ± 0.7 mm, Left: 3.2 ± 0.7 mm) and CKD-II patients (Right: 3.3 ± 0.6 mm, Left: 3.2 ± 0.7 mm; *p* = 0.006 and *p* = 0.028, respectively), suggesting that substantial cortical deterioration occurs primarily in advanced CKD (Table 2). AI demonstrated a dose-response relationship, with progressive reduction from controls (Right: 3.2 ± 0.8 mm, Left: 3.2 ± 0.8 mm) to CKD-II (Right: 3.0 ± 0.7 mm, Left: 3.0 ± 0.7 mm) to CKD-I (Right: 2.7 ± 0.7 mm, Left: 2.7 ± 0.8 mm; *p* = 0.002 and *p* = 0.005, respectively). This gradient effect suggests that cortical thinning in the antegonial region may be a more sensitive indicator of CKD-related mandibular bone deterioration (Table 2). Although not statistically significant, the PMI showed a trend toward higher values in the CKD-I group. Analysis of mandibular cortical morphology revealed significant differences in MCI classification distribution (*p* < 0.001). MCI class I (normal endosteal margin) was observed in only 5.4% of CKD-I patients compared to 35.2% of controls, while MCI class III (porous endosteal margin) was present in 33.9% of CKD-I patients versus 18.2% of controls. This qualitative deterioration complements the quantitative findings from linear measurements, suggesting both architectural and dimensional mandibular changes in CKD. Lamina dura loss was significantly more prevalent in both CKD subgroups (CKD-I: 23.2%, CKD-II: 23.7%) compared to controls (6.8%). Due to sample size limitations affecting the statistical power for rare findings, soft tissue calcifications were analyzed by comparing all CKD patients (*n* = 94) with controls (*n* = 88). This combined analysis revealed significantly higher prevalence of soft tissue calcifications in CKD patients, particularly CAC and sialolithiasis (*p* = 0.009). Pulp chamber size analysis included a subset of patients (CKD-I: *n* = 36, CKD-II: *n* = 26, control: *n* = 66) due to missing mandibular molars. No statistically significant differences were observed between groups, though a trend toward larger horizontal pulp chamber dimensions was noted in CKD-I patients (4.1 ± 0.9 mm) compared to controls (3.8 ± 0.6 mm, *p* = 0.054). These results demonstrate that the CKD patients with eGFRs < 30 mL/min/1.73 m² had more porous bone quality and thinner mandibular cortical layers on CBCT images. Additionally, CBCT image analysis based on the type of KRT received revealed that the AIs of patients undergoing hemodialysis (V001) were statistically significantly lower than those of the control group. Also, the occurrences of MCI class III, loss of lamina dura, and soft tissue calcifications were observed more frequently in patients undergoing hemodialysis (Supplemental 1).

**Table 2:** Comparison of CBCT images between CKD and control groups.

Parameter	Patients with CKD		Control (*n* = 88)	*p*
	CKD-I (*n* = 56)	CKD-II (*n* = 38)		
MI (*M* ± SD)				
Right	2.9 ± 0.7^c,d^	3.3 ± 0.6	3.2 ± 0.7	0.006^a^
Left	2.9 ± 0.7^c^	3.2 ± 0.7	3.2 ± 0.7	0.028^a^
PMI superior (*M* ± SD)				
Right	6.5 ± 2.2	5.7 ± 1.1	5.9 ± 1.6	0.099
Left	6.3 ± 2.2	5.9 ± 1.3	6.0 ± 2.1	0.499
PMI inferior (M ± SD)				
Right	5.4 ± 2.0	4.7 ± 0.9	4.9 ± 1.4	0.296
Left	5.4 ± 1.8	4.9 ± 1.1	5.0 ± 1.7	0.226
AI (*M* ± SD)				
Right	2.7 ± 0.7^c^	3.0 ± 0.7	3.2 ± 0.8	0.002^a^
Left	2.7 ± 0.8^c^	3.0 ± 0.7	3.2 ± 0.8	0.005^a^
Pulp chamber size^e^ (*M* ± SD)				
Vertical	1.5 ± 0.7	1.3 ± 0.5	1.4 ± 0.5	0.469
Horizontal	4.1 ± 0.9^c^	4.0 ± 0.7	3.8 ± 0.6	0.054
MCI, *n* (%)	CKD-I^c^	CKD-II^c^		< 0.001^b^
Class I	3 (5.4)	4 (10.5)	31 (35.2)	
Class II	34 (60.7)	26 (68.4)	41 (46.6)	
Class III	19 (33.9)	8 (21.1)	16 (18.2)	
Lamina dura loss, *n* (%)	CKD-I^c^	CKD-II^c^		0.006^b^
Not present	43 (76.8)	29 (76.3)	82 (93.2)	
Present	13 (23.2)	9 (23.7)	6 (6.8)	
Soft-tissue calcifications, *n* (%)				0.009^b^
Carotid artery calcifications	9 (9.5)		2 (2.3)	
Osteoma cutis	2 (2.1)		3 (3.4)	
Sialolith	7 (7.4)		0 (0)	
Tonsillolith	5 (5.3)		4 (4.5)	
Pulp chamber calcifications	7 (7.4)		3 (3.4)	

M ± SD: mean ± standard deviation; *n* (%): number (%); MI: mental index; PMI: panoramic mandibular index; AI: antegonial index; MCI: mandibular cortical index.

a Statistically significant difference using Kruskal-Wallis test (*p* < 0.05).

b Statistically significant difference using Fisher’s exact test (*p* < 0.05).

c Statistically significant difference (*P* < 0.05), Mann-Whitney test or Fisher’s exact test with Bonferroni post hoc comparison CKD-1 or CKD-II groups compared with the control.

d Statistically significant difference (*p* < 0.05), Mann-Whitney test with Bonferroni post hoc comparison CKD-1 compared with the CKD-II group.

e Several of the subjects were missing due to CBCT reconstruction and missing molars; 36 patients for CKD-I, 26 for CKD-II, and 66 healthy subjects for control groups were included in the analysis.

### Serum levels of the markers related to bone metabolism

Biochemical marker analysis was limited by the retrospective nature of the study, with some serum values unavailable within the 6-month window of CBCT imaging. The number of evaluable patients for each marker is reported in Table 3, with sample sizes ranging from 23 to 173 across different parameters, representing an important limitation. Despite these constraints, biochemical analysis demonstrated significant metabolic disturbances in CKD patients. The levels of serum ALP, P, and PTH intact ECLIA of the CKD-I group were significantly elevated compared to the control group; and the levels in the CKD-II group were intermediate between those of the CKD-I and control groups. Notably, PTH intact ECLIA level in the CKD-I group (250 ± 205.8 pg/mL) was significantly higher than that in the CKD-II group (68.9 ± 71.5 pg/mL) and control group (40.2 ± 17.7 pg/ml, *p* < 0.001). Conversely, serum level of 25(OH)D was statistically significantly higher in the control group (64.7 ± 22.7 ng/mL) compared to both CKD groups (CKD-I, 16.8 ± 11.0; CKD-II, 25.5 ± 11.9 ng/mL, *p* < 0.001) (Table 3). Although 1,25(OH)_2_D level in the CKD groups tended to be lower than in the control group, this level was not in line with the trend of lower 25(OH)D in the CKD-I group. This discrepancy may be attributed to the higher use of calcitriol, vitamin D and its analogs, in CKD-I patients (Table 1). This use was aimed at reducing PTH levels as part of the patient treatment strategy. Additionally, the sample size for 1,25(OH)2D analysis was a limitation in that it was too small to obtain statistical significance.

**Table 3:** Serum level of markers related to bone metabolism.

Parameters	Patients with CKD		Control	*p*
	CKD-I (*n* = 56)	CKD-II (*n* = 38)		
ALP (U/L)	93.3 ± 56.9 (55)^b^	89.1 ± 46.6 (38)^b^	67.5 **±** 17.3 (48)	0.002^a^
Ca (mg/dL)	9.3 ± 1.0 (55)	9.5 ± 0.6 (38)	9.4 **±** 0.5 (80)	0.455
P (mg/dL)	4.4 ± 1.7 (55)^b^	3.6 ± 0.7 (37)	3.5 **±** 0.6 (79)	0.007^a^
PTH Intact ECLIA (pg/mL)	250.1 ± 205.8 (34)^b,c^	68.9 ± 71.5 (28)	40.2 **±** 17.7 (56)	< 0.001^a^
TSH (µlU/m)	3.9 ± 5.7 (19)	2.1 ± 1.3 (21)	2.7 **±** 1.3 (40)	0.263
25(OH)D (ng/ml)	16.8 ± 11.0 (18)^b^	25.5 ± 11.9 (15)^b^	64.7 **±** 22.7 (27)	< 0.001^a^
1,25(OH)_2_D (ng/mL)	46.9 ± 37.9 (3)	44.7 ± 30.5 (5)	63.0 **±** 22.6 (15)	0.486

ALP: alkaline phosphatase; Ca: calcium; P: phosphorus; PTH Intact ECLIA: intact-parathyroid hormone electrochemiluminescence immunoassay; TSH: thyroid-stimulating hormone; 25(OH)D: 25-hydroxy vitamin D; 1,25(OH)_2_D: 1,25-dihydroxy vitamin D.

a Statistically significant difference using Kruskal-Wallis test (*p* < 0.05).

b Statistically significant difference (*p* < 0.05), Mann-Whitney test with Bonferroni post hoc comparison CKD-1 or CKD-II groups compared with the control.

c Statistically significant difference (*p* < 0.05), Mann-Whitney test with Bonferroni post hoc comparison CKD-1 compared with the CKD-II group. Values are presented as mean **±** standard deviation (numbers of subjects analyzed).

### Linear regression analysis of CBCT images

Multivariable linear regression analysis identified significant associations between systemic factors and specific bone abnormalities in CKD patients (Table 4). Elevated ALP levels were positively associated with PMI measurements (PMI superior-Rt, β = 0.010, *p* = 0.005; PMI inferior-Rt, β = 0.010, *p* = 0.003), providing quantitative evidence for the relationship between bone turnover and mandibular morphometry. Female sex emerged as a significant factor for reduced antegonial thickness (AI-£= −0.323, *p* = 0.008) and increased likelihood of advanced cortical deterioration (MCI-I vs. MCI-III, β = 85.399, *p* = 0.002). Age showed the most consistent associations across multiple parameters, correlating with reduced antegonial thickness bilaterally (AI-Rt, β = −0.022, *p* < 0.001; AI-Lt, β = −0.018, *p* < 0.001), increased lamina dura loss (β = 1.051, *p* = 0.032), and progressive cortical deterioration (MCI-I vs. MCI-II, β = 20.511, *p* = 0.012; MCI-I vs. MCI-III, β = 1.275, *p* < 0.001). This suggests that older CKD patients experience compounded effects of age-related and disease-related bone deterioration. The presence of diabetes mellitus specifically influenced cortical morphology (MCI-I vs. MCI-II, β = 20.511, *p* = 0.012), while increased serum creatinine levels – reflecting worsening kidney function – were associated with both reduced antegonial thickness (AI-Rt, β = −0.041, *p* = 0.003; AI-Lt, β = −0.030, *p* = 0.038) and advanced cortical deterioration (MCI-I vs. MCI-III, β = 1.669, *p* = 0.010). Although several biochemical markers showed significant differences between groups (Table 3), only ALP demonstrated independent associations with radiomorphometric indices in the multivariate analysis, suggesting its potential utility as a clinical predictor of mandibular bone quality in CKD patients. Large β coefficients in some regression models (e.g. β = 85.399 for MCI classifications) result from log-odds transformation and baseline frequency differences between groups rather than biological implausibility.

**Table 4:** Linear regression analysis of CBCT images.

Variables	ALP	Ca	*P*	PTH Intact ECLIA	TSH	25(OH)D	1,25(OH)2D
**MI-Rt**	−0.002 (0.258)	−0.077 (0.363)	0.038 (0.466)	−0.000 (0.787)	−0.021 (0.439)	−0.002 (0.506)	−0.013 (0.152)
**MI-Lt**	−0.000 (0.847)	−0.051 (0.519)	−0.008 (0.871)	−0.000 (0.271)	−0.022 (0.441)	0.001 (0.643)	0.002 (0.762)
**PMI superior-Rt**	0.010 (0.003)^a^ 0.010 (0.005)^b^	0.133 (0.498)	−0.144 (0.236)	0.000 (0.692)	0.020 (0.76)	0.009 (0.241)	0.000 (0.993)
**PMI superior-Lt**	0.005 (0.179)	0.156 (0.481)	−0.132 (0.337)	−0.000 (0.895)	−0.005 (0.956)	0.005 (0.601)	0.001 (0.96)
**PMI inferior-Rt**	0.009 (0.002)^a^ 0.010 (0.003)^b^	0.101 (0.557)	−0.127 (0.231)	0.000 (0.755)	0.028 (0.619)	0.008 (0.228)	−0.001 (0.904)
**PMI inferior-Lt**	0.004 (0.228)	0.022 (0.906)	−0.112 (0.326)	0.001 (0.615)	0.006 (0.936)	0.005 (0.594)	0.001 (0.961)
**AI-Rt**	−0.001 (0.538)	0.087 (0.298)	0.000 (0.994)	−0.000 (0.292)	−0.037 (0.231)	0.005 (0.18)	0.006 (0.412)
**AI-Lt**	−0.001 (0.425)	−0.025 (0.773)	0.047 (0.378)	−0.000 (0.413)	−0.023 (0.429)	0.003 (0.322)	−0.000 (0.951)
**Pulp-Vertical**	−0.002 (0.172)	−0.099 (0.169)	−0.006 (0.89)	−0.000 (0.331)	0.064 (0.317)	0.002 (0.514)	0.005 (0.184)
**Pulp-Horizontal**	0.000 (0.808)	0.036 (0.698)	0.004 (0.949)	−0.000 (0.723)	0.030 (0.661)	−0.004 (0.182)	−0.007 (0.221)
**Lamina dura loss**	1.003 (0.559)	1.254 (0.456)	0.833 (0.395)	1.001 (0.324)	0.965 (0.786)	0.988 (0.392)	1.058 (0.145)
**MCI-I vs. MCI-II**	1.007 (0.289)	0.990 (0.972)	1.343 (0.145)	1.007 (0.102)	0.877 (0.554)	0.991 (0.437)	0.992 (0.688)
**MCI-I vs. MCI-III**	1.006 (0.457)	0.812 (0.523)	1.034 (0.890)	1.008 (0.077) 1.007 (0.372)	1.496 (0.105)	1.001 (0.948)	1.020 (0.533)
**Variables**	Exposure year (N code)	Exposure year (V code)	sex	age	DM	serum creatinine	
**MI-Rt**	0.003 (0.871)	−0.000 (0.944)	−0.268 (0.018)^a^ −0.076 (0.638)	−0.008 (0.035)^a^ −0.007 (0.199)	−0.039 (0.761)	−0.027 (0.057) −0.009 (0.626)	
**MI-Lt**	−0.008 (0.573)	−0.002 (0.587)	−0.318 (0.002)^a^ −0.188 (0.186)	−0.007 (0.034)^a^ −0.004 (0.430)	−0.052 (0.665)	−0.015 (0.245)	
**PMI superior-Rt**	0.024 (0.507)	−0.003 (0.773)	0.335 (0.203)	0.017 (0.046)^a^ 0.016 (0.177)	0.173 (0.564)	0.058 (0.096) 0.025 (0.565)	
**PMI superior-Lt**	0.010 (0.783)	0.006 (0.576)	0.169 (0.568)	0.019 (0.044)^a^ 0.019 (0.093)	−0.121 (0.718)	0.045 (0.268)	
**PMI inferior-Rt**	0.015 (0.644)	−0.001 (0.909)	0.230 (0.315)	0.014 (0.054) 0.014 (0.181)	0.094 (0.718)	0.046 (0.131)	
**PMI inferior-Lt**	0.006 (0.84)	0.004 (0.615)	0.232 (0.344)	0.018 (0.023)^a^ 0.007 (0.486)	0.046 (0.868)	0.039 (0.237)	
**AI-Rt**	0.008 (0.577)	−0.002 (0.609)	−0.268 (0.019)^a^ −0.323 (0.008)^b^	−0.017 (<0.001)^a^ −0.022 (<0.001)^b^	−0.085 (0.513)	−0.033 (0.023)^a^ −0.041 (0.003)^b^	
**AI-Lt**	0.019 (0.191)	−0.001 (0.76)	−0.289 (0.013)^a^ −0.251 (0.084)	−0.018 (<0.001)^a^ −0.018 (<0.001)^b^	−0.064 (0.632)	−0.030 (0.034)^a^ −0.030 (0.038)^b^	
**Pulp-Vertical**	0.001 (0.956)	−0.002 (0.723)	−0.117 (0.255)	−0.007 (0.032)^a^ −0.008 (0.182)	0.077 (0.513)	0.001 (0.956)	
**Pulp-Horizontal**	−0.035 (0.095) −0.035 (0.095)	0.005 (0.305)	−0.162 (0.214)	−0.002 (0.638)	0.000 (0.998)	0.004 (0.78)	
**Lamina dura loss**	1.012 (0.794)	1.018 (0.101)	1.110 (0.800)	1.049 (0.003)^a^ 1.051 (0.032)^b^	2.947 (0.011)^a^ 1.046 (0.942)	0.995 (0.923)	
**MCI-I vs. MCI-II**	0.923 (0.264)	1.008 (0.747)	1.027 (0.946)	1.097 (<0.001)^a^ 20.511 (0.012)^b^	4.951 (0.012)^a^ 1.132 (0.002)^b^	1.323 (0.022)^a^ 1.345 (0.087)	
**MCI-I vs. MCI-III**	0.982 (0.803)	0.990 (0.762)	3.385 (0.010)^a^ 85.399 (0.002)^b^	1.170 (<0.001)^a^ 1.275 (<0.001)^b^	5.474 (0.013)^a^ 4.073 (0.278)	1.357 (0.014)^a^ 1.669 (0.010)^b^	

MI: mental index; Rt: Right; Lt: Left; PMI: panoramic mandibular index; AI: antegonial index; MCI: mandibular cortical index; ALP: alkaline phosphatase; Ca: calcium; P: phosphorus; PTH Intact ECLIA: intact-parathyroid hormone electrochemiluminescence immunoassay; TSH: thyroid-stimulating hormone; 25(OH)D: 25-hydroxy vitamin D; 1,25(OH)_2_D: 1,25-dihydroxyvitamin D; Exposure year (N code): the time from CKD (N code) diagnosis to CBT imaging; Exposure year (V code): the time from _receiving_ kidney replacement therapy (V code) to CBCT imaging.

a Statistically significant in univariable linear regression analysis (*p* < 0.05).

b Statistically significant in multivariable linear regression analysis (*p* < 0.05). Values are presented as coefficients (*p*-values).

## Discussion

In this investigation, we sought to identify maxillofacial bone abnormalities in CKD patients using CBCT imaging and to characterize these abnormalities in relation to disease severity and patient demographics. Our results demonstrated that patients with severe renal impairment (eGFR < 30 mL/min/1.73 m²) exhibited significant mandibular bone quality deterioration, manifested as reduced cortical thickness at the mental foramen and antegonial notch regions. These structural changes align with established CKD-MBD pathophysiological mechanisms [3, 15] and correlate with the significantly elevated serum levels of ALP, P, and PTH intact ECLIA observed in our CKD cohort (Table 3). Multivariate linear regression analysis revealed several factors significantly associated with these bone abnormalities, including elevated serum ALP, female sex, advanced age, diabetes mellitus comorbidity, and increased serum creatinine. Additionally, our CBCT assessments detected a higher prevalence of soft tissue calcifications in the craniofacial region among CKD patients. Notably, hemodialysis patients demonstrated particularly pronounced bone deterioration (Supplemental 1), with 93.2% of this subgroup presenting with severely compromised renal function (eGFR < 30 mL/min/1.73 m²) (Table 1). The qualitative and quantitative mandibular regression observed in our study is supported by several preclinical studies. Rodent models of CKD have consistently demonstrated reduced cortical bone mineral density, diminished cortical thickness, and increased trabeculation in the mandible [16]. Furthermore, biomechanical testing has revealed significant compromises in structural properties of mandibular bone in CKD-MBD animal models compared to controls [15]. Recent work by Yamashita et al. provides compelling evidence that CKD adversely affects both the structural integrity and mechanical properties of maxillary and mandibular cortical bone [17]. Collectively, these findings emphasize the critical importance of cautious pre-operative assessment utilizing CBCT imaging for identifying mandibular bone alterations and implementing modified treatment protocols when considering osseointegrated implant therapy in patients with compromised renal function.

To the best of our knowledge, this study is the first to demonstrate statistically significant radiomorphometric differences in CBCT images between CKD patients and healthy controls, with both MIs and AIs significantly lower in the CKD group. Previous CBCT studies in CKD patients mainly verified qualitative bone changes, specifically porous trabecular bone patterns. Çağlayan et al. [18] found no differences in radiomorphometric indices, but noted variations in MCI, lamina dura loss, and soft tissue calcification – observations consistent with our findings. Similarly, Ersu et al. [19] reported decreased fractal dimension values without significant radiomorphometric differences, while Bilgili and Üçok [20] observed lower trabecular spacing values in CKD patients. Our study advances this research through methodological improvements: a substantially larger sample size (94 CKD patients vs. 15–22 previously), stricter exclusion criteria, and standardized CBCT protocols. Additionally, we uniquely examined correlations between radiological findings and bone metabolism markers. The significant correlation between elevated ALP levels and cortical thinning (PMI superior-Rt, β = 0.010, *p* = 0.005; PMI inferior-Rt, β = 0.010, *p* = 0.003) offers important insight into CKD-related bone deterioration mechanisms. This finding extends beyond previous qualitative studies and aligns with established CKD-MBD pathophysiology [21, 22], potentially offering clinicians a valuable predictive tool for mandibular bone assessment in this patient population.

The clinical implications of our findings are particularly relevant for implant dentistry. Dental implant treatment relies on the osseointegration of the implant fixture with the surrounding bone and ankylosis at the bone-implant interface to support functional masticatory loads [23]. Although studies have indicated favorable outcomes in terms of implant installation and healing among patients with CKD [5, 6], there remains a lack of consensus regarding the safety of implant therapy in patients with CKD-MBD because alterations in bone metabolism may impact the integration and stability of dental implants. There have been several studies that addressed concerns associated with bone healing or dental implant surgery in patients with CKD. One study investigating extraction socket healing at seven- and 60-days post-tooth extraction found that both mineral content and bone thickness in the plane of the x-ray beam were significantly lower in CKD patients and those who had undergone kidney transplantation compared to control subjects. This highlights the necessity for careful consideration before recommending dental implant rehabilitation in this population [24]. In a uremic mouse model, histomorphometric analysis of experimental implants inserted into the femurs of CKD mice indicated a trend of decreased bone-implant contact ratio after 2 weeks of healing. Additionally, the strength of bone-implant integration, assessed via push-in methods, was significantly lower in the CKD group at 2 weeks; but comparable integration levels were achieved by 4 weeks [25]. Certain studies suggest that additional adjuvant therapies such as calcium and vitamin D supplementation and FGF23 neutralization may enhance the efficacy of dental implant treatment [26–28]. In this study, bone abnormalities were more pronounced in CKD patients with an eGFR < 30 mL/min/1.73 m² (CKD-I); and their presence was correlated with elevated serum creatinine and ALP levels, reflecting that bone abnormalities were more pronounced in patients with substantial declines in renal function. While these findings do not demonstrate a definitive impact on implant treatment success or long-term stability in CKD patients, they suggest that increased caution is warranted when conducting these procedures, particularly in patients with progressively declining kidney function.

This study showed an interesting clinical finding that patients receiving hemodialysis had more pronounced bone abnormalities. This result aligns closely with previous findings that demonstrate a high incidence of bone fractures predominantly in hemodialysis patients [29, 30]. A retrospective Korean population-based study explored the epidemiology of fractures in patients undergoing KRT and highlighted that hemodialysis patients had the highest incidence rate of fractures and kidney transplant patients had the lowest incidence [31]. This increased fracture risk in hemodialysis patients was associated with several factors, including time after KRT initiation, advanced age, comorbidities such as diabetes mellitus and liver cirrhosis, and metabolic disturbances like elevated PTH levels and chronic acidosis [31]. Some of these contributing factors are consistent with our findings. In our study, the increase in bone abnormalities was associated with several factors, including elevated ALP levels, advanced age, female sex, presence of diabetes mellitus, and serum creatinine levels. However, no association was demonstrated with time after CKD diagnosis or after receiving KRT or with other bone metabolism marker levels except those of ALP. The difference in the findings may be due to the relatively small number of patients analyzed in this study. Therefore, further research is needed to clarify the mechanisms and clinical implications of bone abnormalities in patients with CKD, especially research with larger cohorts and longitudinal studies.

The significantly increased prevalence of soft tissue calcifications in CKD patients, particularly CACs and sialolithiasis, reflects the systemic nature of mineral metabolism disorders in this population. These findings are consistent with the well-established propensity for ectopic calcification in CKD due to dysregulated calcium-phosphate homeostasis and secondary hyperparathyroidism [2, 3]. From a clinical standpoint, CBCT examinations performed for dental treatment planning could provide opportunistic screening for extracranial calcifications, potentially facilitating early identification of cardiovascular risk factors in CKD patients. This dual diagnostic capability – assessing both mandibular bone quality and detecting systemic calcifications – may enhance the clinical value of CBCT examinations in CKD patients. However, appropriate protocols for medical referral should be established when such calcifications are detected during routine dental imaging.

This study has several important limitations. The retrospective design precluded standardization of the timing between biochemical testing and CBCT imaging, though we limited this interval to 6 months. The cross-sectional nature prevents assessment of temporal relationships between biochemical changes and bone deterioration. The study was limited to mandibular bone assessment due to more standardized anatomical landmarks and greater measurement reproducibility compared to the maxilla’s variable anatomy and sinus proximity. However, maxillary and mandibular bones may respond differently to CKD-related metabolic disturbances, warranting future comprehensive evaluation of both jaws. Additionally, pulp chamber size analysis was limited by reduced sample size due to missing mandibular molars, which may have limited statistical power to detect meaningful changes in dental hard tissue morphology related to CKD. Furthermore, while we did not perform formal intra-observer reliability testing, several methodological strengths mitigate potential measurement bias: 1) complete examiner blinding to group assignment with randomized image presentation; 2) standardized protocols based on established Ledgerton’s classification; 3) rigorous quality assurance with weekly device calibration; and 4) between-group differences exceeding typical measurement variability. This blinded assessment approach prevents systematic measurement bias and is more critical than random measurement error for valid between-group comparisons. Future prospective studies with standardized protocols, reliability assessments, and longitudinal follow-up are needed to better understand bone change progression in CKD patients.

In conclusion, CBCT analysis revealed specific quantitative changes in CKD patients, with significantly reduced MI and AI values, particularly in those with eGFR <30 mL/min/1.73 m². Our findings suggest that CBCT-derived radiomorphometric indices can serve as complementary tools to biochemical markers like ALP and creatinine when evaluating mandibular bone quality in CKD patients. Clinicians should consider comprehensive CBCT evaluation before implant placement in patients with advanced renal impairment or those undergoing hemodialysis, as these patients demonstrated significant cortical thinning that may affect implant stability. However, these recommendations should be interpreted within the constraints of our retrospective, single-center study design without longitudinal follow-up. The cross-sectional nature of our analysis limits our ability to establish temporal relationships between biochemical changes and bone deterioration. Future prospective studies with standardized protocols are needed to validate these findings and determine their long-term clinical significance for dental treatment planning in the CKD population.

## Supplementary Material


