# Supplementary material for: Evaluation of mandibular bone abnormalities in patients with chronic kidney disease using cone beam computed tomography: A retrospective study
Source: Acta Odontol Scand. 2025 Sep 3;84:44619. doi: 10.2340/aos.v84.44619 (PMC12416334; doi:10.2340/aos.v84.44619)
Supplement: Supplementary file 1 [file AOS-84-44619-s1.pdf]

Supplementary material has been published as submitted. It has not been copyedited or typeset by Acta Odontologica Scandinavica.

Supplementary Information

Supplemental 1. The characteristics of CBCT images in patients receiving kidney replacement therapy.

	Patients with CKD				Control (n = 88)	p-value
	V001 (n = 44)	V0003 (n = 7)	V0005 (n = 17)	Non-KRT (n = 26)		
MI (M ± SD)						
Right	2.93± 0.11	3.14±0.13	3.21± 0.30	3.28±0.13	3.2±0.7	0.151
Left	2.89± 0.09	3.27± 0.21	2.93± 0.17	3.15± 0.16	3.2±0.7	0.095
PMI superior (M ± SD)						
Right	6.47± 0.33	5.61± 0.24	6.34± 0.44	5.72± 0.28	5.9±1.6	0.282
Left	6.50± 0.30	5.42± 0.34	5.73± 0.50	6.11± 0.33	6.0±2.1	0.237
PMI inferior (M ± SD)						
Right	5.39± 0.30	4.78± 0.25	5.25± 0.39	4.67±0.22	4.9±1.4	0.510
Left	5.51± 0.26	4.62± 0.30	5.17± 0.39	4.97± 0.27	5.0±1.7	0.182
AI (M ± SD)						
Right	2.78± 0.10 [c)]	3.07± 0.16	3.03± 0.17	2.70± 0.16	3.2±0.8	0.007 [a)]
Left	2.77± 0.11	2.74± 0.29	3.05± 0.08	2.76± 0.15	3.2±0.8	0.035 [a)]
Pulp chamber size [d)] (M ± SD)						
Vertical	1.55± 0.12	1.36± 0.18	1.32± 0.12	1.31±0.09	1.4±0.5	0.836
Horizontal	4.08± 0.14	4.31± 0.23	3.94± 0.17	4.01± 0.14	3.8±0.6	0.190
MCI, n (%)	V0001 [c)]	V0003	V0005	Non-KRT [c)]		0.001 [b)]
Class I	3 (6.8)	0 (0.0)	2 (4.5)	1 (3.8)	31 (35.2)	
Class II	28 (63.6)	5 (71.4)	13 (29.5)	15 (57.7)	41 (46.6)	
Class III	13 (29.5)	2 (28.6)	2 (4.5)	10 (38.5)	16 (18.2)	
Lamina dura loss, n (%)	V0001 [c)]	V0003	V0005	Non-KRT		0.010 [b)]
Not present	31 (70.5)	6 (85.7)	14 (82.4)	21 (80.8)	82 (93.2)	
Present	13 (29.5)	1 (14.3)	3 (17.6)	5 (19.2)	6 (6.8)	
Soft-tissue calcifications, n (%)	V0001 [c)]	V0003	V0005	Non-KRT		0.010 [b)]
carotid artery calcifications	7 (15.9)	0 (0.0)	0 (0.0)	2 (7.7)	2 (2.3)	
osteoma cutis	1 (2.3)	0 (0.0)	0 (0.0)	1 (3.8)	3 (3.4)	
Sialolith	4 (9.1)	0 (0.0)	2 (11.8)	1 (3.8)	0 (0)	
Tonsillolith	1 (2.3)	2 (28.6)	1 (5.9)	1 (3.8)	4 (4.5)	
Pulp chamber calcifications	3 (6.8)	2 (28.6)	3 (17.6)	2 (7.7)	3 (3.4)	

[a)] Statistically significant difference using Kruskal-Wallis test ($p < 0.05$). [b)] Statistically significant difference using Fisher's exact test ($p < 0.05$). [c)] Statistically significant difference ($p < 0.05$), Mann-Whitney test or Fisher's exact test with Bonferroni post hoc comparison patients coded with V001, V003, V005 and non-KRT compared with the control group. [d)] Statistically significant difference (p 0.05), Mann-Whitney test with Bonferroni post hoc comparison CKD-1 compared with the CKD-II group. [e)] Several of the subjects were missing due to CBCT reconstruction and missing molars, so 30 patients for V0001, five

for V0003, 13 for V0005, 14 for non-KRT, and 66 healthy subjects for control groups were included in the analysis. M ± SD: mean ± standard deviation, n (%): number (%), MI: mental index, PMI: panoramic mandibular index, AI: antegonial index, MCI: mandibular cortical index.
